# TRAIL-Mediated Apoptosis in Breast Cancer Cells Cultured as 3D Spheroids

**DOI:** 10.1371/journal.pone.0111487

**Published:** 2014-10-24

**Authors:** Siddarth Chandrasekaran, Jocelyn R. Marshall, James A. Messing, Jong-Wei Hsu, Michael R. King

**Affiliations:** Department of Biomedical Engineering, Cornell University, Ithaca, New York, United States of America; University of Michigan, United States of America

## Abstract

TNF-alpha-related-apoptosis-inducing-ligand (TRAIL) has been explored as a therapeutic drug to kill cancer cells. Cancer cells in the circulation are subjected to apoptosis-inducing factors. Despite the presence of these factors, cells are able to extravasate and metastasize. The homotypic and heterotypic cell-cell interactions in a tumor are known to play a crucial role in bestowing important characteristics to cancer cells that leave the primary site. Spheroid cell culture has been extensively used to mimic these physiologically relevant interactions. In this work, we show that the breast cancer cell lines BT20 and MCF7, cultured as 3D tumor spheroids, are more resistant to TRAIL-mediated apoptosis by downregulating the expression of death receptors (DR4 and DR5) that initiate TRAIL-mediated apoptosis. For comparison, we also investigated the effect of TRAIL on cells cultured as a 2D monolayer. Our results indicate that tumor spheroids are enriched for CD44^hi^CD24^lo^ALDH1^hi^ cells, a phenotype that is predominantly known to be a marker for breast cancer stem cells. Furthermore, we attribute the TRAIL-resistance and cancer stem cell phenotype observed in tumor spheroids to the upregulation of cyclooxygenase-2 (COX-2)/prostaglandin E2 (PGE_2_) pathway. We show that inhibition of the COX-2/PGE_2_ pathway by treating tumor spheroids with NS-398, a selective COX-2 inhibitor, reverses the TRAIL-resistance and decreases the incidence of a CD44^hi^CD24^lo^ population. Additionally, we show that siRNA mediated knockdown of COX-2 expression in MCF7 cells render them sensitive to TRAIL by increasing the expression of DR4 and DR5. Collectively, our results show the effect of the third-dimension on the response of breast cancer cells to TRAIL and suggest a therapeutic target to overcome TRAIL-resistance.

## Introduction

In the hematogenous metastatic cascade, cells from the primary tumor enter the peripheral circulation after which they can mimic the leukocyte adhesion cascade to extravasate through the blood vessel wall and establish in a secondary site [1]. While cancer cells are in the circulation, they are subjected to apoptosis-inducing signals from immune cells such as natural killer cells that elicit an anti-tumor response [2]. Despite the presence of apoptosis-inducing agents, cancer cells can metastasize, causing 90% of cancer related deaths [3]. Cancer therapy is entering a paradigm shift from radiation and broad-spectrum chemotherapeutic agents to less hazardous directed molecules that can specifically target cancer cells. TRAIL is one such molecule that plays a key role in body's natural defense mechanism, which is currently being studied in the field of cancer therapy [4]–[6]. TRAIL-mediated apoptosis is initiated by the binding of TRAIL to death receptors (DR4 and DR5), which induces the formation of the death-inducing signaling complex (DISC) [7]. The surface expression of death receptors plays a key role in transmitting the apoptosis-inducing signal. Several cancer cell lines have been shown to be resistant to TRAIL-mediated apoptosis by decreasing the expression of death receptors [8], internalizing death receptors by constitutive endocytosis [9], upregulating anti-apoptotic proteins such as Bcl-2 [10], activating cellular survival pathways such as PI3K/Akt signaling pathway [11], upregulating decoy receptors [12], [13], or downregulating pro-apoptotic proteins such as Caspase 8 [14]. Thus, studying the underlying mechanism behind TRAIL-resistance exhibited by certain cancer cells could lead to more effective use of TRAIL in anti-cancer therapy.

Cell-cell interactions in primary tumors have been shown to play a significant role in determining the fate of a cell that leaves the primary site and enters the peripheral circulation [15]. Though cancer cell lines serve as a good model for studying different aspects of the metastatic cascade, physiologically relevant interactions may be lost in 2D monolayer culture [16]. The dimensionality of the system used to study cancer has an important role in studying several aspects of cancer biology. For instance, multicellular 3D tumor spheroids have been shown to be resistant to drugs and radiation [17]. The third dimension is also implicated in the presence of cancer stem cells within solid tumors [18], [19]. We have previously demonstrated an *in vitro* cell culture method using polydimethylsiloxane (PDMS) coated multiwell plates to propagate cell lines as 3D spheroids [20]. This method has been used for the enrichment of a cancer stem cell subpopulation in the WM115 melanoma cell line [21]. We have also shown that breast cancer cell lines cultured as 3D tumor spheroids on PDMS exhibit increased adhesion to E-selectin and have more migratory and invasive properties [22], [23].

In primary tumors, the relatively poor circulatory network often results in a hypoxic zone of oxygen-deprived cancer cells [24]. Hypoxic conditions are known to trigger the expression of transcription factors termed hypoxia-inducible factors (HIF-1α and HIF-1β). HIFs have several downstream targets that can be activated to further facilitate tumor progression [25]. Our recent work indicates that tumor spheroids cultured on PDMS are hypoxic and they express HIF-1α and HIF-1β [22]. The most important downstream effector of hypoxia inducible factors is cyclooxygenase-2 (COX-2) [26]. COX-2 is a protein involved in the biosynthesis pathway of a class of lipid signaling molecules called prostaglandins [27]. COX-2-induced prostaglandin E2 (PGE_2_) expression has several consequences in tumor progression. PGE_2_ secreted by tumor cells and/or stromal cells can induce drug resistance, migration, and invasion of cancer cells, to name a few [27]. It has been shown that COX-2 expression can downregulate the expression of death receptor in cancer cells rendering them resistant to TRAIL-mediated apoptosis [28], [29]. However, the effect of hypoxia-induced COX-2 expression on inducing TRAIL resistance is unknown. The present study investigates the effect of the third dimension in cancer cell responsiveness to TRAIL. To demonstrate this, we cultured breast cancer cell lines as tumor spheroids on PDMS and dissociated them to explore their response to TRAIL-mediated apoptosis. This is relevant because the cells that enter the peripheral circulation have just transitioned from 3D to 2D form and are available for therapeutic targeting in the bloodstream. This work was undertaken to investigate the molecular basis of TRAIL resistance exhibited by certain cancer cells in the peripheral circulation.

## Materials and Methods

### Cell lines and culture conditions

Breast cancer cell lines BT20 (ATCC, HTB-19) and MCF7 (ATCC, HTB-24) were cultured in ATCC-formulated Eagle's Minimum Essential Medium (EMEM) (ATCC, 30-2003) supplemented with 10% fetal bovine serum (FBS) (Atlanta Biologicals, S11050H), and 1% penicillin/streptomycin (PS) (Invitrogen, 15140-122). The media for MCF7 was additionally supplemented with 0.01 mg/mL of bovine insulin (Sigma Aldrich, I1882-100 MG). Cell lines were maintained at 37°C with 5% CO_2_. For propagating cells as 3D spheroids on PDMS coated 24-well plate, we followed a protocol described previously [22]. Briefly, 50,000 cells were seeded in a single well of a 24-well plate coated with PDMS. Cells were cultured as tumor spheroids for 48 h before being used for any of the assays/treatment conditions in this study.

### TRAIL and NS-398 treatment

Cells propagating as tumor spheroids were collected by pooling the media from 24-well plates. Since tumor spheroids were loosely adhered to the underlying PDMS substrate, removal of media enabled the collection of tumor spheroids. The pooled media was centrifuged at 1000 rpm for 10 min and the resulting cell pellet was dissociated in 1 mL of trypsin-EDTA and incubated for 15 min at 37°C. An equivalent amount of cell culture media was added to neutralize the effect of trypsin and the cells were centrifuged and resuspended in 1 mL of complete cell culture media and plated in 24-well plates at a seeding density of 50,000 cells per well. The cells were cultured for 6 h to account for the effect of trypsin on cell surface receptors. After 6 h, the media was exchanged with fresh media with recombinant human TRAIL (R&D Biosystems, 357-TL/CF) at a concentration of 200 ng/mL. After 24 h of culture, cells were visualized for apoptotic cells using bright field phase contrast microscopy on Olympus IX81 microscope. We followed a similar protocol for treating monolayer cells with TRAIL by collecting monolayer cells from T-25 flasks. NS-398 (Sigma, N194) was reconstituted in DMSO at a concentration of 80 mM. The cells were simultaneously treated with 100 µM of NS-398 and 200 ng/mL of TRAIL. The cells were also treated with 100 µM NS-398 to determine its effect of on cell viability.

### Cell viability assays

Cell viability assays were conducted to evaluate the effect of TRAIL and NS-398 on cells cultured as tumor spheroids and monolayers. A standard colorimetric MTT assay was performed after 24 h in culture to determine the percentage of viable cells. We added 3-(4, 5-Dimethylthiazol-2-yl)-2, 5-diphenyltetrazolium bromide) (Chemicon (Millipore) CT0-A) to detect mitochondrial activity. The absorbance was measured at 690 nm using a microplate reader (Bio-Tek Instruments). The absorbance was normalized with respect to untreated control and the viability reported as percentages. To numerically quantify the percentage of cells undergoing apoptosis we used the Annexin V-FITC and Propidium Iodide (PI) apoptosis detection kit (Trevigen, 4380-01-K). We followed the protocol provided by the manufacturer for flow cytometry-based detection of apoptotic cells using a Guava easyCyte flow cytometer (Millipore).

### Flow cytometry and fluorescence-activated cell sorting

The expression levels of TRAIL receptors DR4 and DR5 were investigated on cells propagating as a 2D monolayer on tissue culture plates and 3D spheroids on PDMS. To prepare cells propagating as spheroids on PDMS for flow cytometry, we dissociated tumor spheroids as described and cultured them for 6 h in complete cell culture media before performing flow cytometry. The cells were collected using enzyme-free cell dissociation buffer and then centrifuged at 1000 rpm for 10 min. Approximately, 1×10^6^ cells were resuspended in 100 µL of staining solution composed of 1% bovine serum albumin (BSA) in 1x phosphate buffer saline (PBS). Cells propagating as a monolayer were subjected to the same protocol and resuspended in 100 µL of staining buffer. The cells were then incubated at 4°C in the dark with PE-conjugated mouse anti-human DR4 and DR5 (eBioscience, 12-6644-42 and 12-9908-42) for 45 min at the manufacturer recommended volume. PE mouse IgG isotype control (eBioscience, 12-4714-42) was used to account for nonspecific interaction of antibodies with the cells. The cells were washed twice in PBS and reconstituted in PBS at a concentration of 500 cells/µL to be analyzed using a Guava easyCyte flow cytometer. To determine the expression of stem cell markers CD44, CD24 and ALDH1 in cells propagating as tumor spheroids and monolayers we used FITC-conjugated mouse anti-human CD44 (BD Biosciences, 555478), PE-conjugated mouse anti-human CD24 (BD Biosciences, 555428), and their corresponding isotype controls (BD Biosciences, 555742 and 555574). After staining for cell surface CD44 and CD24, the cells were washed twice in PBS and resuspended in PBS for analysis/sorting or fixed and permeabilized for staining intracellular protein ALDH1. The cells were fixed using 2% paraformaldehyde solution for 30 min at 4°C. The fixed cells were then permeabilized using 0.2% Tween 20 at 37°C for 10 min. The permeabilized cells were washed in PBS containing 2% FBS and then incubated with mouse anti-human ALDH1 or corresponding isotype control (Santa Cruz Biotechnology, sc-166362 and sc-3879) at 4°C for 45 min. The cells were then washed in PBS and incubated with APC conjugated anti-mouse IgG secondary antibody (BioLegend, 405308). After incubation, the cells were washed twice in PBS and reconstituted in 1x PBS at a concentration of 500 cells/µL to be analyzed using a Guava easyCyte flow cytometer.

For sorting CD44^hi^CD24^lo^ cells from tumor spheroids, the cells were stained using FITC conjugated CD44 and PE conjugated CD24 antibodies. After staining, the cells were washed twice in PBS resuspended in cell culture media and sorted using a BD Biosciences FACS-Aria flow sorter. The post-sort purity was verified after sorting and the cells were cultured in serum-free cell culture media overnight and used for further analysis. During sorting experiments CD44^hi^CD24^lo^ cells were collected in one sample tube and the rest of the cells (non-stem like cells) were collected in a separate sample tube.

### Western blotting and ELISA

The expression of COX-2, DR4 and DR5 proteins in monolayer and spheroids were detected by standard western blotting protocol. Briefly, cell lysates were separated by SDS-PAGE and transferred to a nitrocellulose blotting membrane (Bio-Rad, 162-0115). Membranes were blocked with 5% milk for 60 min at room temperature (RT, 25°C). The membranes were then incubated overnight at 4°C with 1∶2000 mouse anti-human COX-2 specific antibody (BD Biosciences, 610204), and 1∶2000 mouse anti-human DR4 and DR5 (BioLegend, 307201 and 307302), in 5% non-fat dry milk. Membranes were also probed with mouse anti-human β-actin (Santa Cruz biotechnology, sc-81178) at 1∶2000 dilution as a loading control. Following the overnight incubation with primary antibody, the membranes were washed with tris-buffered saline (TBS) with 0.1% Tween-20 and then incubated for 60 min at RT with 1∶1000 anti-mouse IgG-HRP (BD Biosciences, 554002) in 5% non-fat dry milk. The bands were visualized using a chemiluminiscent HRP substrate (Millipore, WBKL 50500) in a luminescent image analyzer (Fujifilm, LAS-4000). The level of PGE_2_ in the cell culture media conditioned by cells propagating as tumor spheroids and monolayers was determined using Prostaglandin E_2_ human ELISA kit (Invitrogen, KHL1701) following a protocol supplied by the manufacturer.

### Confocal microscopy

For death receptor and EpCAM labeling, monolayer cells and spheroid cells were removed from the 24-well plate using enzyme-free dissociation buffer to preserve the presence of membrane proteins. Cells were then resuspended at a concentration of 1×10^6^ cells/mL in 1% BSA. 15,000 cells were deposited onto a microscope slide using a Shandon Cytospin 3 (Harlow Scientific). The cell coated slides were then fixed and in 4% paraformaldehyde for 30 min at room temperature of 25°C (RT). Slides were blocked for 1 h at RT with 1% BSA. Slides were incubated in antibody solution containing primary antibodies against DR4, DR5 and EpCAM followed by incubation with secondary antibody solution conjugated to Alexa Fluor 594 for death receptor and FITC for EpCAM. The cells were then incubated with 50 µL of NucBlue fixed cell probe (LifeTechnologies, R37606) to stain the nucleus. Slides were imaged using Zeiss 710 laser scanning confocal microscope.

### Small interfering RNA (SiRNA) transfection and real-time polymerase chain reaction (RT-PCR) analysis

MCF7 cells were seeded in 6-well plates in media without antibiotics until they reached 40% confluence. Cells were transfected on the following day using Lipofectamine 2000 (Invitrogen, 11668-019). 300 pmol Cox-2/control siRNA (Santa Cruz Biotechnology, sc-29279 and sc-37007) was separately premixed with Lipofectamine 2000 for 20 min, and then applied to cells. 6 h following transfection, media was replaced with complete cell culture media. Protein levels were assessed by RT-PCR 24 h following transfection. Total RNA was isolated using Trizol (Invitrogen, 15596-026) and reverse transcription was performed using RNA to cDNA EcoDry Premix (Clontech, 639282). 10 ng of cDNA produced by the reverse transcription of total RNA was used in each quantitative PCR reaction. Also included in the 15 µL qPCR reaction system were 7.5 µL iQ SYBR Supermix (Bio-Rad, 170-8882), 1 µL of 2 µM forward primer and 1 µL of 2 µM reverse primer and nuclease free water. The expression level of human COX-2 gene was normalized to the expression level of the housekeeping gene GAPDH. The primer sequences (Integrated DNA Technologies) of COX-2 are:

Forward: 5′-CCAGCACTTCACGCATCAG-3′.

Reverse: 5′-CAGACCAGGCACCAGACC-3′.

The primer sequences of GAPDH are:

Forward: 5′-AGAGCACAAGAGGAAGAGAGAGAC-3′.

Reverse: 5′-AGCACAGGGTACTTTATTGATGGT-3′.

### Statistical analysis

MTT viability assay was performed on cells seeded in five different wells of a 24-well plate for each treatment condition (n = 5). The qPCR and ELISA experiments were done in triplicate (n = 3). The statistical significance was tested using unpaired student t-test and the value of p is displayed in the figure where applicable.

## Results

### BT20 and MCF7 spheroids show reduced expression of death receptors and are more resistant to TRAIL-mediated apoptosis

**Figure 1:**
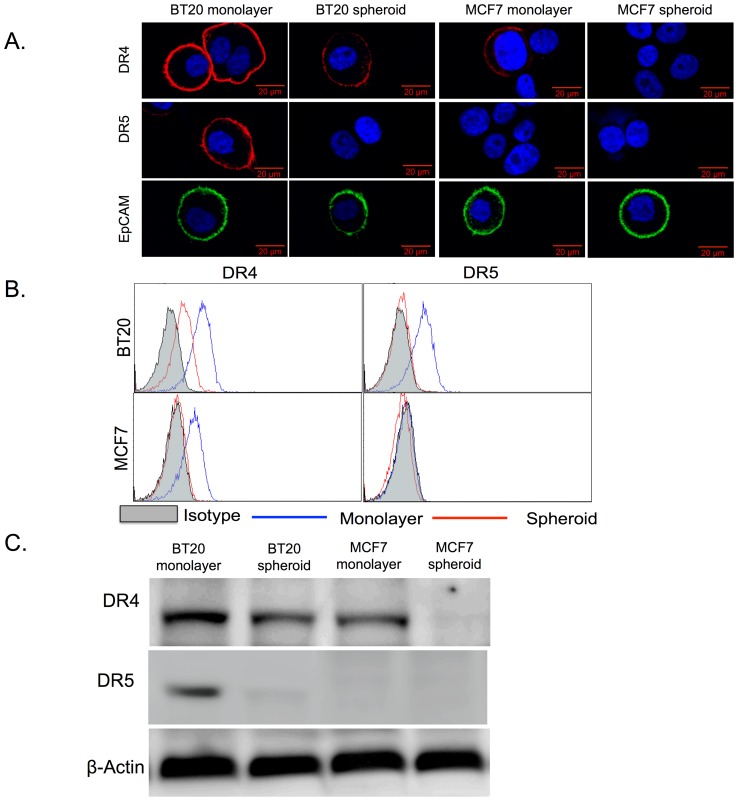
BT20 and MCF7 spheroids have a lower expression of death receptors. (A) Confocal micrographs of BT20 and MCF7 cells cultured as 3D spheroids and 2D monolayer stained with antibodies against DR4 (red), DR5 (red) and EpCAM (green). (Scale bar  = 20 µm) (B) Flow cytometry histograms comparing DR4 and DR5 expression in BT20 and MCF7 spheroids and monolayer. (C) Western blot analysis of total DR4 and DR5 expression in cell lysates from BT20 and MCF7 spheroids and monolayer.

**Figure 2:**
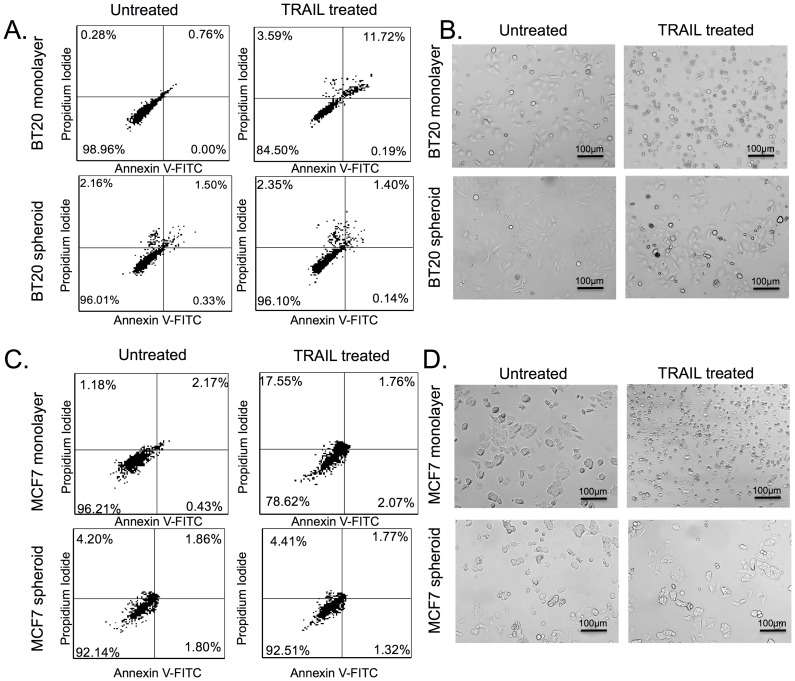
BT20 and MCF7 spheroids are more resistant to TRAIL-mediated apoptosis. Flow cytometry Annexin-V/Propidium Iodide scatterplots of untreated and 200 ng/mL of TRAIL treated (A) BT20 and (B) MCF7 cells cultured as 3D spheroids and 2D monolayer 24 h after treatment conditions. Cells can be classified into four groups based on dye uptake: viable cells (lower left quadrant), early apoptotic cells (lower right quadrant), late apoptotic cells (upper right quadrant) and necrotic cells (upper left quadrant). Bright field images of untreated and 200 ng/mL TRAIL treated (C) BT20 and (D) MCF7 cells from spheroid and monolayer culture after 24 h after treatment conditions.

BT20 and MCF7 spheroids expressed lower levels of death receptors (DR4 and DR5) in comparison to monolayer cells (Fig. 1). Confocal micrographs indicated a strong staining for surface death receptor expression in BT20 (DR4 and DR5) and MCF7 cells (DR4 only) cultured as a monolayer and a weak or no staining in BT20 (weak for DR4 and no staining for DR5) and MCF7 (no staining for either DR4 or DR5) cells cultured as 3D tumor spheroids (Fig. 1A). We also stained the cells for EpCAM expression and the confocal micrographs showed that there was no change in surface expression of EpCAM when cells were cultured as 3D spheroids (Fig. 1A). Flow cytometry analysis revealed that BT20 cells cultured as a monolayer expressed ∼5.4 fold higher DR4 when compared to BT20 cells cultured as spheroids (Fig. 1B). BT20 cells cultured a monolayer expressed DR5 whereas BT20 cells cultured as spheroids did not express DR5 (Fig. 1B). Similarly, MCF7 monolayer cells expressed DR4 whereas MCF7 spheroid cells did not express DR4 (Fig. 1B). MCF7 cells did not express DR5 (Fig. 1B). Western blot analysis of whole cell lysate confirmed our flow cytometry results (Fig. 1C). BT20 and MCF7 cells cultured as tumor spheroids were more resistant to TRAIL-mediated apoptosis when compared to BT20 and MCF7 cells cultured as a monolayer. Flow cytometry scatterplots from apoptosis detection assay showed that 3.42% of BT20 cells and 5.60% of MCF7 cells cultured as tumor spheroids were in late apoptosis stage (upper right quadrant) as opposed to 14.34% of BT20 cells and 15.93% of MCF7 cells cultured as a monolayer (Fig. 2A and 2B). Note that BT20 and MCF7 cells are resistant to TRAIL and they become more resistant when cultured in 3D. The bright field images also indicate that monolayer cells treated with 200 ng/mL of TRAIL show morphological changes characteristic of apoptosis (more rounded and suspended) whereas cells cultured as tumor spheroids were more resistant to TRAIL-mediated apoptosis (Fig. 2C and 2D) with most of the cells viable (spread out and adherent) after TRAIL treatment. Untreated BT20 and MCF7 cells cultured as a monolayer and tumor spheroids are shown for comparison (Fig. 2C and 2D).

### BT20 and MCF7 spheroids show increased expression of breast cancer stem cell markers

**Figure 3:**
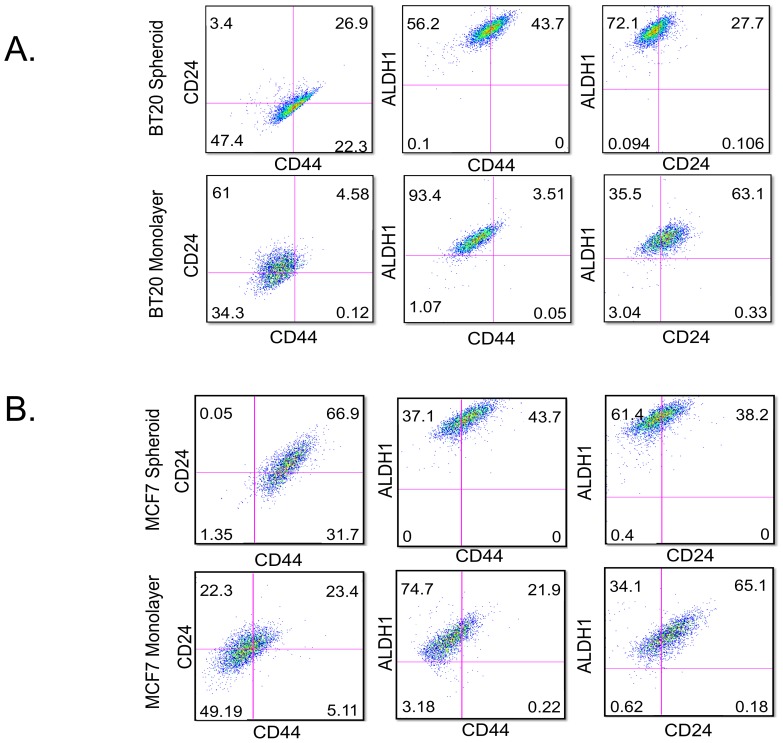
BT20 and MCF7 spheroids have increased incidence of cells with breast cancer stem cell phenotype. Flow cytometry histograms indicating the expression of CD44, CD24 and ALDH1 in (A) BT20 spheroid and monolayer cells and (B) MCF7 spheroid and monolayer cells.

BT20 and MCF7 cells cultured as tumor spheroids were enriched for CD44^hi^CD24^lo^ALDH1^hi^ cells in comparison to cells cultured as a monolayer (Fig. 3). BT20 cells cultured as spheroids had 22.3% CD44^hi^CD24^lo^ cells as opposed to 0.12% of CD44^hi^CD24^lo^ cells in BT20 cells cultured as a monolayer (Fig. 3A). BT20 spheroids also showed a higher level of ALDH1 activity in comparison to BT20 monolayer. The ALDH1^hi^ cells were predominantly CD44^hi^CD24^lo^ in BT20 spheroids (43.7% of CD44^hi^ALDH1^hi^ cells and 72.1% of CD24^lo^ALDH1^hi^ cells). In comparison, ALDH1^hi^ cells were predominantly CD44^l^°CD24^hi^ in BT20 monolayer. Taken together, these results indicate that BT20 cells cultured as spheroids show increased expression of breast cancer stem cell markers. A similar trend was observed for MCF7 cells cultured as spheroids. MCF7 spheroids had 31.7% of CD44^hi^CD24^lo^ cells as opposed to 5.11% of CD44^hi^CD24^lo^ cells in MCF7 monolayer (Fig. 3B). Similarly, MCF7 spheroids had a higher level of ALDH1 activity and most of the ALDH1^hi^ cells had a D44^hi^CD24^lo^ phenotype (Fig. 3B).

### CD44^hi^CD24^lo^ cells isolated from BT20 tumor spheroids do not express DR4

**Figure 4:**
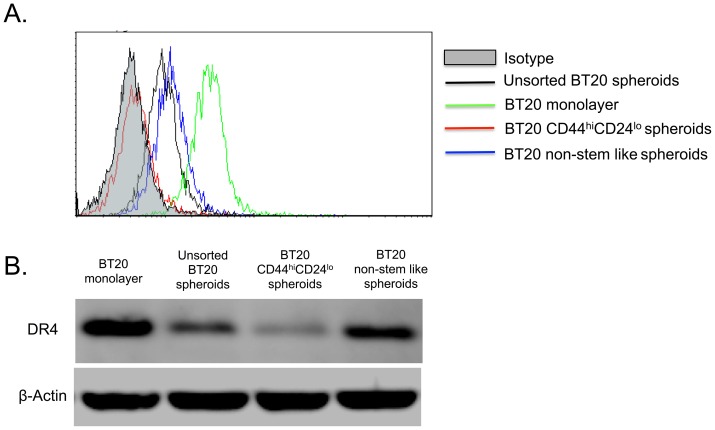
CD44^hi^CD24^lo^ BT20 spheroid cells have a lower expression of DR4 in comparison to non-stem like cells. (A) Confocal micrographs of FACS sorted CD44^hi^CD24^lo^ cells from BT20 and MCF7 spheroids stained with FITC conjugated CD44 antibody and PE conjugated CD24 antibody (Scale bar  = 50 µm) (B) Bright field images of CD44^hi^CD24^lo^ and non-stem like cells from BT20 and MCF7 spheroids (C) Flow cytometry histograms comparing the expression of death receptors in FACS sorted cells and monolayer cells.

Given the prevalence of CD44^hi^CD24^lo^ cells in BT20 and MCF7 cells cultured as spheroids and since most of the CD44^hi^CD24^lo^ cells showed high ALDH1 activity, we isolated CD44^hi^CD24^lo^ BT20 cells cultured as 3D tumor spheroids using fluorescence activated cell sorting. The CD44^hi^CD24^lo^ cells were collected in one collection tube and the rest of the cells were collected as a separate population and are termed non-stem like cells. Flow cytometric analysis of CD44^hi^CD24^lo^ cells revealed that they had no detectable expression of DR4 to initiate TRAIL-mediated apoptosis (Fig. 4A). The expression of DR4 was slightly higher in non-stem like cells in comparison to unsorted BT20 spheroid cells. Note that BT20 cells cultured as tumor spheroids do not express DR5 (Fig. 1) and MCF7 spheroids do not express either DR4 or DR5 (Fig. 1). Thus the lower expression of DR4 in BT20 tumor spheroids is a synergistic effect of the lack of DR4 expression in CD44^hi^CD24^lo^ and DR4 expression in non-stem like cells. Western blotting of whole cell lysate confirmed the flow cytometry observations (Fig. 4B). BT20 cells cultured as a monolayer showed the maximum expression of DR4, followed by non-stem like cells sorted from 3D spheroids, unsorted spheroid cells and CD44^hi^CD24^lo^ spheroid cells in decreasing order.

### COX-2/PGE_2_ pathway is upregulated in tumor spheroids

**Figure 5:**
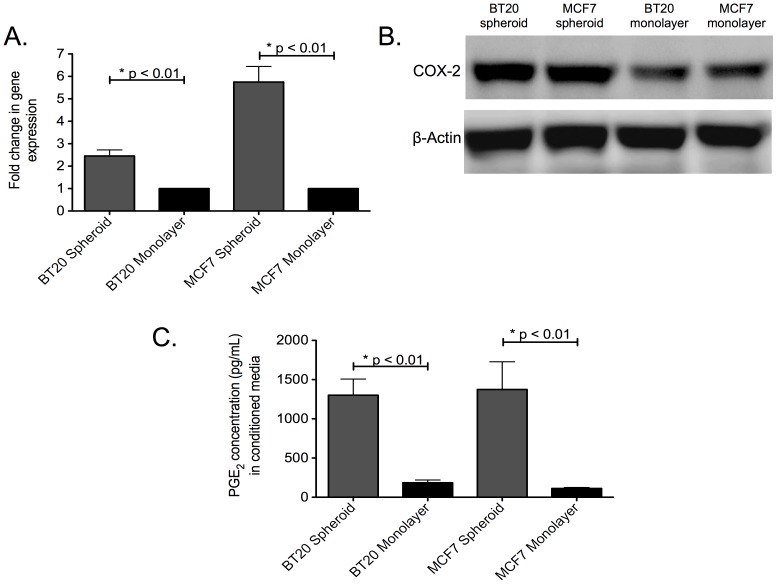
The COX-2/PGE_2_ pathway is upregulated in BT20 and MCF7 spheroids. (A) qPCR data showing fold change in COX-2 gene expression in BT20 and MCF7 spheroids in comparison to monolayer cells (n = 3) (B) Western blot data indicating the expression of COX-2 protein in whole cell lysates from BT20 and MCF7 cells cultured as spheroids and monolayer (C) ELISA results for PGE_2_ level in media conditioned by BT20 and MCF7 cells cultured as spheroids and monolayer (n = 3).

Recently, we showed that BT20 and MCF7 spheroids are hypoxic and express the hypoxia inducible factors HIF-1α and HIF-1β [22]. One of the direct downstream targets of HIF-1α and HIF-1β is COX-2. RT-PCR analysis revealed ∼2.2 and ∼5.4 fold increased gene expression of COX-2 in BT20 and MCF7 cells cultured as spheroids in comparison to their monolayer counterparts (Fig. 5A). Western blotting of whole cell lysate revealed that BT20 and MCF7 spheroids had more COX-2 protein when compared to BT20 and MCF7 monolayer (Fig. 5B). Assaying media conditioned by BT20 and MCF7 cells cultured as spheroids and monolayer for PGE_2_ levels indicated a significant increase in tumor spheroid conditioned media (1301.33 pg/mL and 1374.377 pg/mL in BT20 and MCF spheroid conditioned media, respectively) in comparison to media conditioned by monolayer cells (184.36 pg/mL and 112.465 pg/mL in BT20 and MCF monolayer conditioned media, respectively) (Fig. 5C).

### COX-2 inhibitor reverses TRAIL resistance in BT20 and MCF7 cells and reduces the incidence of stem cell phenotype in tumor spheroids

**Figure 6:**
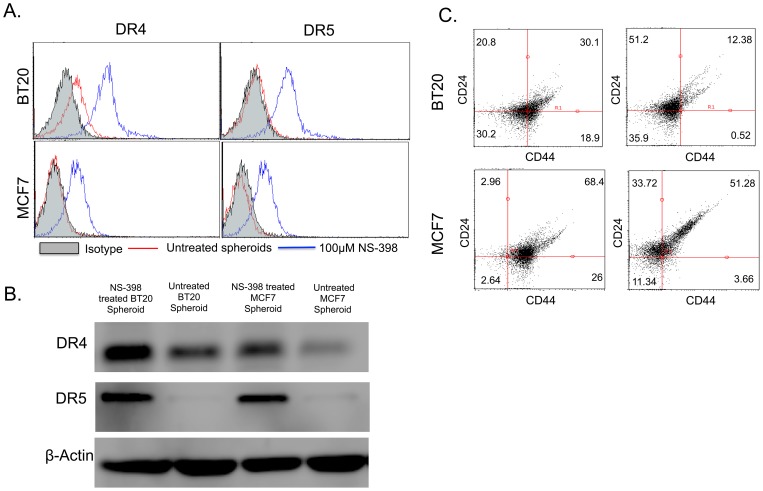
COX-2 inhibitor treatment upregulates the expression of DR4 and DR5 in BT20 and MCF7 spheroids. (A) Flow cytometry histograms showing the effect of 100 µM NS-398 treatment on death receptor expression in BT20 and MCF7 cells cultured as spheroids. (B) Western blot analysis of whole cell lysates from untreated and NS-398 treated BT20 and MCF7 spheroids for DR4 and DR5 expression. (C) Flow cytometry scatter plots showing the effect of NS-398 treatment on the prevalence of CD44^hi^CD24^lo^ population in BT20 and MCF7 cells cultured as spheroids.

**Figure 7:**
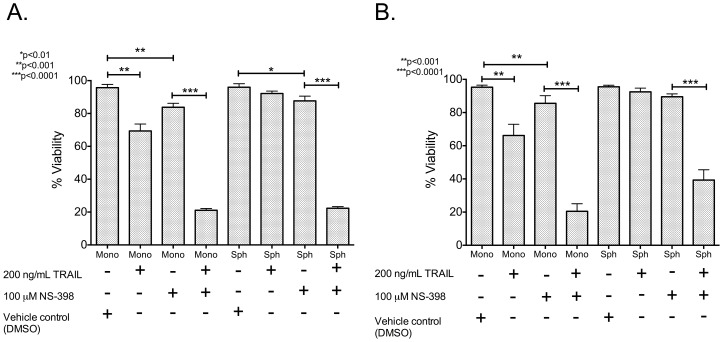
COX-2 inhibitor treatment sensitizes BT20 and MCF7 spheroid and monolayer cells to TRAIL-mediated apoptosis. MTT assay results quantifying the viability of BT20 and MCF7 cells under different treatment conditions (n = 5).

Since the COX-2/PGE_2_ pathway is upregulated in BT20 and MCF7 spheroids, the effect of COX-2 inhibitor in reversing the observed TRAIL-resistance and stem cell phenotype in tumor spheroids was investigated. BT20 and MCF7 spheroids were treated with NS-398, a COX-2 specific inhibitor. NS-398 treatment was found to increase the expression of DR4 and DR5 in BT20 spheroids and MCF7 spheroids (Fig. 6A and 6B). Flow cytometry histograms indicate an increase in the expression of DR4 and DR5 in spheroid grown cells (Fig. 6A) upon NS-398 treatment. Western blotting was used to confirm the results obtained from flow cytometry (Fig. 6B). NS-398 treatment also decreased the incidence of CD44^hi^CD24^lo^ cells in BT20 and MCF7 spheroids (Fig. 6C). NS-398 treated BT20 and MCF7 spheroids had 0.52% and 3.66% of CD44^hi^CD24^lo^ cells, respectively, in comparison to 18.9% and 26% of CD44^hi^CD24^lo^ cells, respectively, in untreated samples (Fig. 6C). MTT assay revealed that combined NS-398 and TRAIL treatment significantly reduced the viability of BT20 and MCF7 spheroids (Fig. 7). The assay was normalized with respect to DMSO treated control (vehicle for NS-398). BT20 spheroids were sensitized to TRAIL-mediated apoptosis when treated with NS-398 (Fig. 7A). Similarly, MCF7 spheroids were also sensitized to TRAIL-mediated apoptosis when treated with NS-398 (Fig. 7B). NS-398 treatment alone for 24 h had a significant effect on the viability BT20 cells cultured in monolayer form and spheroid form, and MCF7 cells cultured as a monolayer (Fig. 7). However, the viability was drastically reduced when cells were subjected to combined treatment with NS-398 and TRAIL. ELISA analysis of media conditioned by NS-398 treated BT20 and MCF7 spheroids for PGE_2_ concentration revealed that PGE_2_ levels were lower than the detection limit of the kit (31.3 pg/mL).

### siRNA-mediated knockdown of COX-2 sensitizes MCF7 cells to TRAIL-mediated apoptosis

**Figure 8:**
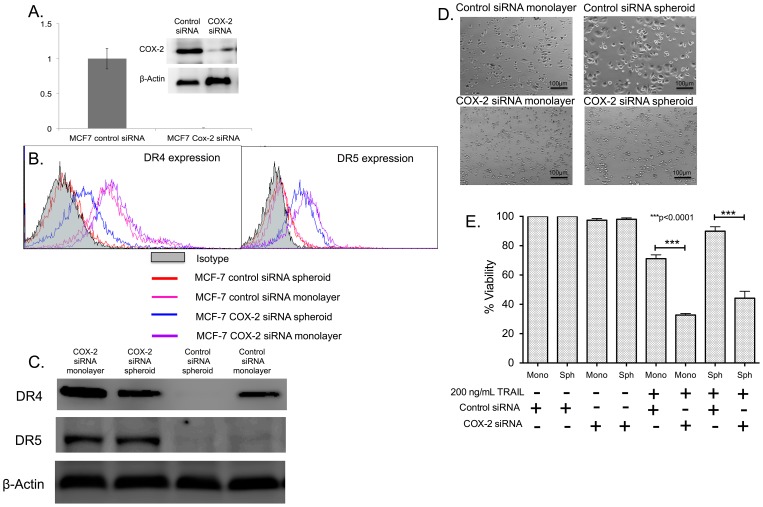
COX-2 knockdown reverses TRAIL-resistance in MCF7 spheroid and monolayer cells. (A) qPCR and western blot data showing the relative expression of COX-2 gene (n = 3) and COX-2 protein in control siRNA and COX-2 siRNA transfected MCF7 cells. (B) Flow cytometry histograms and (C) western blot analysis comparing the expression of DR4 in MCF7 cells transfected with control siRNA and COX-2 siRNA cultured as monolayer and spheroids. (D) Bright field images and (E) MTT assay results (n = 5) quantifying the effect of 200 ng/mL of TRAIL in control siRNA and COX-2 siRNA transfected cells cultured as monolayer and spheroids.

Since MCF7 spheroids had ∼5.4 fold increased COX-2 gene expression (Fig. 5A), we knocked down the expression of COX-2 in MCF7 cells using siRNA against COX-2. RT-PCR results revealed that there was a complete knockdown of COX-2 gene expression in cells transfected with COX-2 siRNA in comparison to cells transfected with control siRNA (Fig. 8A). Western blot analysis revealed reduced expression of COX-2 protein in transfected cells (Fig. 8A). COX-2 siRNA transfected cells indicated a significant upregulation of surface DR4 and DR5 expression in both monolayer and spheroid culture (Fig. 8B). However, cells transfected with control siRNA showed no changed in the expression of surface DR4 or DR5 when cultured as tumor spheroids (Fig. 8B). Western blot analysis also revealed upregulation of DR4 and DR5 in MCF7 cells transfected with COX-2 siRNA when cultured as a monolayer and tumor spheroids (Fig. 8C). COX-2 and control siRNA transfected MCF7 cells were then treated with 200 ng/mL TRAIL. Bright field images indicated that control siRNA treated cells cultured as a monolayer were sensitive to TRAIL-mediated apoptosis whereas cells cultured as tumor spheroids were resistant to TRAIL-mediated apoptosis (Fig. 8D). MCF7 cells transfected with COX-2 siRNA were the most susceptible to TRAIL-mediated apoptosis in both monolayer and spheroid culture conditions (Fig. 8D). MTT assay further revealed a significant reduction in cellular viability in MCF7 cells transfected with COX-2 siRNA after 24 h of TRAIL treatment (Fig. 8E). The effect of COX-2 mediated downregulation of DR4 and DR5 in 3D is lost in siRNA-transfected cells, which is evident by similar sensitivity to TRAIL-mediated apoptosis in transfected cells irrespective of monolayer or spheroid culture (Fig. 8E). Assaying the media conditioned by COX-2 siRNA transfected MCF7 spheroids for PGE_2_ level revealed that it was below the detection limit of 31.3 pg/mL.

## Discussion

It has been estimated that one million cells are shed per gram of tumor per day into the peripheral circulation [30]. If the majority of these cells possessed the capacity to metastasize, metastatic disease would be unavoidable. However, hematogenous metastasis is considered to be a relatively inefficient process because the fluid shear stress [31], [32] and natural killer cell mediated response [33] can result in the death of the majority of cancer cells present in the circulation. Despite the presence of these factors, cancer cells do in fact metastasize and 90% of all cancer related deaths are attributed to metastases at distant sites. Natural killer cells in the circulation are known to induce cytotoxicity in cancer cells in circulation and this is primarily mediated by TRAIL [34]. TRAIL has been investigated as a potential therapeutic agent because of its ability to induce apoptosis in cancer cells expressing death receptors that initiate TRAIL-mediated apoptosis and has an advantage over other drugs used for targeting cancer cells because it is a molecule involved in body's natural defense mechanism [13], [35]–[37]. It has been shown that sensitivity to TRAIL is anchorage-dependent [38], [39] suggesting that anchorage-independent circulating tumor cells may have altered response to TRAIL in comparison to anchorage-dependent cells from the primary tumor. Hence, it is important to study and understand the underlying mechanism behind TRAIL-resistance exhibited by cancer cells in the circulation.

We have developed an *in vitro* cell culture technique to propagate cancer cells as tumor spheroids on PDMS [20]–[22]. The hydrophobic PDMS hinders cells adhering to the substrate and favors cell-cell adhesion favoring the propagation of cells as 3D spheroids. The pathophysiological gradients in a primary tumor are more closely recapitulated in a tumor spheroid, making them a useful platform for investigating different aspects of the metastatic cascade. The pathophysiological gradients in 3D melanoma spheroid model have been shown to induce a TRAIL-resistant phenotype in melanoma spheroids [40]. The 3D skin-melanoma model was shown to be resistant to TRAIL in comparison to 2D culture and combination treatment with ultra-violet radiation or cisplatin rendered spheroids sensitive to TRAIL [40]. We have previously shown that BT20 and MCF7 cells form tightly packed spheroids on PDMS [22]. Cells that are shed from the primary tumor have left the 3D microenvironment which is known to play an important role determining the fate of the cell in circulation such as their ability to survive apoptosis-inducing signals from circulating immune cells. To mimic this physiological scenario, BT20 and MCF7 cells were cultured as tumor spheroids and then dissociated to investigate their response to TRAIL. These two cell lines are of particular interest because we recently showed that BT20 and MCF7 spheroids had increased invasiveness and interaction with E-selectin that mediate the hematogenous metastatic cascade [22]. MCF7 cells have shown to be resistant to TRAIL-mediated apoptosis [41] and studies have been focused on sensitizing MCF7 cells to TRAIL-mediated apoptosis [42], [43]. BT20 cells have also been shown to be resistant to TRAIL owing to a lower degree of activation of pro-apoptotic caspases upon TRAIL treatment [44]. Thus BT20 and MCF7 cells have a TRAIL-resistant phenotype and we investigated if cells cultured as 3D spheroids possess a more TRAIL-resistant phenotype.

BT20 and MCF7 spheroids had a lower expression of the surface death receptors that initiate TRAIL-mediated apoptosis (Fig. 1), they were more resistant to TRAIL, and showed increased viability when compared to cells cultured as a monolayer (Fig. 2). The apoptosis assay is based on the ability of Annexin-V (early apoptosis detection) to bind to exposed phospholipids of the inner leaflet of the plasma membrane. Phosphatidylserine (PS) is translocated from the inner to the outer membrane of a cell in early stages of apoptosis. Annexin-V is a protein that can bind (conjugate to PS) and a cell that is positive for Annexin-V is considered to be in early stages of apoptosis. Propidium Iodide (PI) is a membrane-impermeable DNA intercalating agent, generally excluded from viable cells. Late apoptotic cells and necrotic cells with compromised plasma membrane can be stained with PI. A cell that is positive for both Annexin-V and PI is considered to be in late stages of apoptosis and a cell that is positive for only PI is considered to be necrotic. Our results revealed that the percentage of TRAIL-treated monolayer cells in the later stages of apoptosis was significantly more in comparison to TRAIL-treated spheroid cells (Fig. 2A and C).

Primary tumors are known to be heterogeneous with subpopulations of cells with different properties. Cancer stem cells are a subpopulation of cells with the ability to self-renew and differentiate into different types of cancer cell [45]. There are distinct markers to identify cancer stem cells in several types of cancers. CD44^hi^CD24^lo^ALDH1^hi^ cells are known to be markers for cancer stem cells in breast cancer [46], [47]. CD44 is a glycoprotein that mediates the interaction of cancer cells to E-selectin expressed on endothelial cells. CD24 is also a cell adhesion protein that mediates cell-extracellular matrix interactions. ALDH1 is an enzyme that is a functional marker for adult stem cells and high activity of ALDH1 has been used to identify breast cancer stem cells. We showed tumor spheroids were enriched for cells with breast cancer stem cell phenotype (CD44^hi^CD24^lo^ALDH1^hi^) (Fig. 3). Cancer cells with a stem cell phenotype are considered to be more resistant to conventional chemotherapy owing to their expression of multi-drug resistant receptors [48]–[50]. Our results showed that CD44^hi^CD24^lo^ cells isolated from BT20 spheroids do not express DR4 to initiate TRAIL-mediated apoptosis (Fig. 4). The lower DR4 expression in BT20 spheroids is a combined result of non-stem like cells expressing DR4 and CD44^hi^CD24^lo^ cells lacking DR4 expression.

The hypoxic microenvironment in a primary tumor results in the expression of hypoxia inducible factors, a set of transcription factors that have been shown to drive the growth of slow dividing subpopulations of hypoxic cells with cancer stem cell phenotype [51]. One of the important downstream targets of hypoxia inducible factor is COX-2. COX-2 overexpression has been previously shown to downregulate the expression of death receptors in colon cancer cells [29] and favor chemoresistance exhibited by MCF7 cells [52]. COX-2 inhibition has been shown to increase death receptor expression and make hepatocellular cancer cells susceptible to TRAIL-mediated apoptosis [28], [53]. However, to date, the underlying mechanism of COX-2 mediated downregulation of death receptor remains unknown [29]. Recently, PGE_2_ expression by stromal cells has been shown to induce stem cell phenotype in breast cancer [54]. However, PGE_2_ alone cannot induce stem cell phenotype and IL-6 secreted by tumor-derived fibroblasts play a key role in the expansion of aggressive stem cell subpopulation [54]. It has been show that PGE_2_ in combination with IL-6 secreted by tumor-derived fibroblast can increase the incidence of stem-like cells in breast cancer cells [54]. We identified that the COX-2/PGE_2_ pathway was upregulated in tumor spheroids (Fig. 5). The basal expression of COX-2 in BT20 and MCF7 monolayers (Fig. 5B) could possibly explain the TRAIL-resistant phenotype of BT20 and MCF7 cells. Our results show very low response to a high concentration of TRAIL (200 ng/mL) in BT20 and MCF7 monolayer cells (∼15–19% cell death), indicative of a TRAIL-resistant phenotype. Upregulation of COX-2 in tumor spheroids could further enhance the TRAIL resistance in BT20 and MCF7 spheroids. NS-398, a COX-2 inhibitor was able to reverse TRAIL-resistance and stem cell phenotype in BT20 and MCF7 spheroids (Fig. 6). NS-398 has been previously shown to selectively inhibit COX-2 expression in cancer cells [52], [55]–[57]. Combined treatment with NS-398 and TRAIL was able to induce apoptosis in BT20 and MCF7 spheroid and monolayer cells by upregulating the expression of death receptor (Fig. 7). siRNA-mediated knockdown of COX-2 drastically altered the responsiveness of MCF7 cells to TRAIL (Fig. 8). COX-2 siRNA-transfected MCF7 cells were more sensitive to TRAIL than control siRNA transfected MCF7 cells, which confirms the role of COX-2 in inducing TRAIL-resistant phenotype.

**Figure 9:**
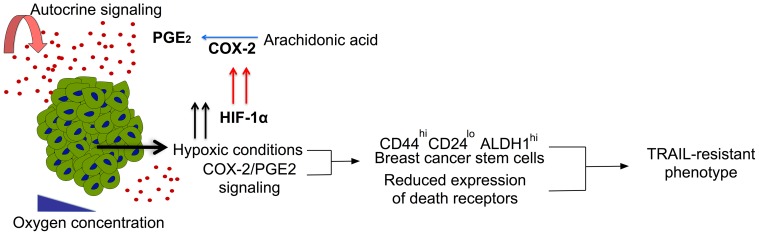
The proposed mechanism behind TRAIL-resistance in tumor spheroids. Hypoxic conditions trigger HIF-1α, upregulating the expression of COX-2, which is involved in the biosynthesis pathways of PGE_2_ from Arachidonic acid. Hypoxic conditions and PGE_2_ signaling together favor cells with breast cancer stem cell phenotype and lowers the expression of surface death receptors conferring resistance to TRAIL-mediated apoptosis in 3D.

Identifying molecular targets to overcome drug resistance can be of great significance in cancer therapy. Recently, the NF-κB pathway was identified as a molecular target responsible for stem cell phenotype and drug resistance in pancreatic, lung and breast cancer [58]. The paradoxical cell-cell interactions and several factors in the tumor microenvironment can result in constant evolution of cell subpopulations resistant to conventional therapies designed to target the tumor [15]. Engineering host immune cells to target cancer cells are becoming more popular because of their inherent ability to protect host cells [59], [60]. This has led to several studies investigating the potential of augmenting the therapeutic efficacy of immune cells such as T-cells [61], NK cells [62], dendritic cells [63] etc. TRAIL ligand secreted by NK cells and T-cells, is known to preferentially kill cancer cells. Normal non-transformed cells are resistant to TRAIL [64], making it an attractive candidate for therapeutic use. We recently showed the potential application of TRAIL functionalized liposomes to augment immune cells in the circulatory [65] and lymphatic systems [66] to target cancer cells in the circulation. Thus, it is important to investigate the TRAIL-resistant phenotype of cancer cells in circulation. To mimic the physiological process we cultured cancer cells as 3D spheroids and dissociated them to evaluate their response to TRAIL-mediated apoptosis. We showed that BT20 and MCF7 spheroids become more resistant to TRAIL when grown in 3D spheroid form. Recently we showed that BT20 and MCF7 cells cultured as tumor spheroids on PDMS expressed HIF-1α and HIF-1β [22]. Hypoxia-inducible factors regulate COX-2/PGE_2_ pathway in cancer cells [67] and cancer stem cell maintenance [54]. Our results indicate that knocking down the expression of COX-2 by either using a COX-2 selective inhibitor or COX-2 siRNA, reversed TRAIL-resistant phenotype and the incidence of cells with stem cell phenotype in breast cancer spheroids. Taken together, this suggests that hypoxia induced COX-2/PGE_2_ upregulation in 3D could render cells more resistant to TRAIL-mediated apoptosis by lowering the expression of death receptors. The COX-2/PGE_2_ signaling pathway along with hypoxic conditions in 3D may also favor a subpopulation of cells with cancer stem cell phenotype that is more resistant to TRAIL than non-stem like cells (Fig. 9). Thus, combination therapy using COX-2 inhibitor and TRAIL may be used as a treatment strategy for targeting TRAIL-resistant cells in circulation.

## Conclusions

The majority of currently available data on TRAIL-resistance exhibited by cancer cell lines is based on conventional 2D monolayer cell culture. However, primary tumors exist in a 3D microenvironment that allows for cell-cell interactions in physiologically relevant gradients in factors such as glucose, oxygen and growth factors. As a result, 3D cell culture for cancer has gained significant attention in the past decade to investigate several facets of tumor progression such as invasiveness, resistance to drugs, the presence of cancer stem cells, etc. The dimensionality of a primary tumor can be replicated *in vitro* using a range of cell culture platforms that are currently being used for 3D cell culture. Our approach is a simple one based on the use of biocompatible PDMS polymer to propagate cancer cells as 3D spheroids. In this work, we showed that combination treatment using a COX-2 selective inhibitor and TRAIL could significantly reduce the viability of TRAIL-resistant breast cancer cells.
